# Asiatic Acid Protects against Doxorubicin-Induced Cardiotoxicity in Mice

**DOI:** 10.1155/2020/5347204

**Published:** 2020-05-15

**Authors:** Xiaoping Hu, Baijun Li, Luocheng Li, Bowen Li, Jinlong Luo, Bin Shen

**Affiliations:** ^1^Department of Cardiovascular Surgery, Renmin Hospital of Wuhan University, Wuhan, Hubei 430060, China; ^2^Department of Thoracic Cardiovascular Surgery, The People's Hospital of Guangxi Zhuang Autonomous Region, Nanning, Guangxi Zhuang Autonomous Region, China

## Abstract

The use of doxorubicin (DOX) can result in depression of cardiac function and refractory cardiomyopathy. Currently, there are no effective approaches to prevent DOX-related cardiac complications. Asiatic acid (AA) has been reported to provide cardioprotection against several cardiovascular diseases. However, whether AA could attenuate DOX-related cardiac injury remains unclear. DOX (15 mg/kg) was injected intraperitoneally into the mice to mimic acute cardiac injury, and the mice were given AA (10 mg/kg or 30 mg/kg) for 2 weeks for protection. The data in our study found that AA-treated mice exhibited attenuated cardiac injury and improved cardiac function in response to DOX injection. AA also suppressed myocardial oxidative damage and apoptosis without affecting cardiac inflammation in DOX-treated mice. AA also provided protection in DOX-challenged cardiomyocytes, improved cell viability, and suppressed intracellular reactive oxygen species (ROS) in vitro. Detection of signaling pathways showed that AA activated protein kinase B (AKT) signaling pathway in vivo and in vitro. Furthermore, we found that AA lost its protective effects in the heart with AKT inactivation. In conclusion, our results found that AA could attenuate DOX-induced myocardial oxidative stress and apoptosis via activation of the AKT signaling pathway.

## 1. Introduction

Anthracyclines are the primary choice particularly in patients with severe leukemias, lymphomas, and solid tumors [1]. Cardiotoxicity is a fatal side effect of doxorubicin (DOX), which largely limits its clinical use. The use of DOX can also trigger cardiac arrhythmia, pericarditis, depression of cardiac function, and refractory cardiomyopathy in a dose-dependent manner [2, 3]. Moreover, previous studies demonstrated that cardiac dysfunction even occurs at a very low therapeutic dose of DOX [4, 5]. DOX-related cardiac injury is irreversible, and currently, there are no effective approaches to prevent DOX-related cardiac complications in cancer patients with chemotherapy. Therefore, it is of great importance to find drugs that could protect against DOX-induced cardiac injury.

DOX-induced cardiotoxicity involves in multiple biological processes including increased reactive oxygen species (ROS) production and lipid peroxidation, which eventually lead to the death of cardiomyocytes. DOX treatment resulted in massive production of superoxide anion free radicals (O2) and ROS and thereafter caused DNA damage and apoptosis [6, 7]. A previous study found that oxidative stress and subsequent lipid peroxidation could be detected in DOX-treated hearts even at three hours after DOX administration [8]. Therefore, prevention of oxidative stress may be a promising method against DOX-induced cell loss and cardiac dysfunction.

Asiatic acid (AA) is a pentacyclic triterpene in *Centella asiatica*, which has been widely used in China and in India [9]. Previous studies indicated that AA possessed a wide variety of pharmacological effects. AA has been reported to attenuate hepatic ischemia/reperfusion injury, protect against pressure overload-induced cardiac hypertrophy, and reduce cardiovascular remodeling in rats with L-NAME-induced hypertension [10–12]. AA has been reported to activate the cellular antioxidant system to protect rat hepatocyte against carbon tetrachloride-induced injury [13]. Moreover, in vitro data suggested that AA could enhance the nuclear factor erythroid 2-related factor 2 (Nrf2) signal [14], which plays a key role against oxidative injury. However, the effects of AA on DOX-induced cardiotoxicity and the precise mechanisms still remain unclear. In this study, we determined the effect of AA on DOX-induced cardiotoxicity and investigate the underlying mechanisms. Results of our study indicate that DOX-induced cardiac injury can be reduced by AA administration with concomitant activation of protein kinase B (AKT).

## 2. Materials and Methods

### 2.1. Animals and Treatment

All animal studies followed the protocols approved by the Animal Care and Use Committee of Renmin Hospital of Wuhan University (Wuhan, China). All male C57BL/6 mice (male, age: 10 weeks, body weight: 24-26 g) were purchased from HFK Bioscience (Beijing, China). All the mice were housed in a specific pathogen-free facility. DOX (purity ≥ 98.5% as determined by high-performance liquid chromatography) was purchased from Sigma-Aldrich (St. Louis, MO, USA). DOX (15 mg/kg) dissolved into 200 *μ*l isotonic phosphate-buffered saline was injected intraperitoneally into the animals. And the mice in the control group received the same volume of vehicle as a control. The dose of DOX was determined according to a previous study [15]. One week before DOX injection, mice were orally given AA (10 mg/kg or 30 mg/kg) or the same volume vehicle for 2 weeks; this regimen provided one week of AA pretreatment prior to DOX exposure. The dose of AA used in this study was determined according to a previous study [10]. Alteration in body weight was recorded daily during the whole experimental period. Adenovirus vectors carrying dominant-negative AKT (Ad-dnAKT) and GFP (Ad-GFP) were generated by Vigene Biosciences (Rockville, MD, USA). To confirm whether AKT activation was involved in the protective effect of AA, the animals were intramyocardially injected with 1 × 10^9^ viral genome particles (Ad-dnAKT or Ad-GFP) in the left ventricle at one week before DOX injection.

### 2.2. Echocardiography and Hemodynamics

Mice were anesthetized with 1.5% isoflurane, and cardiac function of mice after DOX injection was monitored by the Vevo 770 high-resolution microimaging system (VisualSonics, Toronto, Canada) with an RMV 707-B Scanhead (frequency 30 MHz, focal length 12.7 mm) as previously described [16, 17]. To detect hemodynamics, we used a microtip catheter transducer (Millar, SPR-1000) to insert into the right carotid artery and advanced into the left ventricle, and the obtained data were analyzed by the PowerLab software (ADInstruments, LabChart 5) [15].

### 2.3. Cardiac Injury Detection

Blood was collected from the retroorbital plexus after the mice were anesthetized at three days after DOX injection. After that, plasma samples were isolated via centrifuging whole blood at 1000 g for 15 min. Mouse cardiac troponin I (cTnI) was detected using an ELISA kit from Life Diagnostics, Inc. (PA, USA) according to the manufacturer's protocol. Creatine kinase (CK) and lactate dehydrogenase (LDH) detection kits were purchased from Nanjing Jiancheng Institute of Biotechnology (Nanjing, China). Plasma CK and LDH detection were performed according to the manufacturer's protocol.

### 2.4. Determination of Lipid Peroxidation Levels

The hearts in the indicated groups were excised and homogenized in PBS containing 1% protease inhibitor and 1% phosphatase inhibitor (Thermo Scientific). The level of 4-hydroxynonenal (4-HNE) adduct was determined using a 4-HNE assay kit (Abcam, ab238538, Cambridge, UK). The level of 3-nitrotyrosine (3-NT) was detected using a 3-NT ELISA kit (Abcam, ab116691). For each determination, 100 mg of protein was used.

### 2.5. Detection of Cellular Antioxidant System

The activity of superoxide dismutase (SOD) and the levels of malondialdehyde (MDA) and glutathione (GSH) in the hearts were detected according to the manufacturer's instructions using kits obtained from Nanjing Jiancheng Institute of Biotechnology.

We detected intracellular ROS and superoxide using spectrophotometry with 2′,7′-dichlorodihydrofluorescein diacetate (DCFH-DA) and dihydroethidium (DHE), respectively. In brief, the cells were reacted with 5 *μ*mol/l dyes for 25 mins at 37°C in the dark. After that, the fluorescence intensity of cells was determined using a microplate reader (BioTek Instruments, Inc., VT, USA).

### 2.6. Western Blot

Protein lysates were extracted from frozen heart samples using RIPA buffer supplemented with 1% protease inhibitor and 1% phosphatase inhibitor (Thermo Scientific). A BCA Protein Assay Kit was used to determine the protein concentrations of the heart samples. Thereafter, the proteins were electrophoresed by SDS-PAGE and then transferred to PVDF membranes [18]. After being blocked with 5% nonfat milk for 2 h at room temperature, these proteins were blotted with the following primary antibodies: rabbit anti-Nrf2 (Abcam, ab62352, 1 : 1000), rabbit anti-GAPDH (Abcam, ab181602, 1 : 5000), rabbit anti-Bax (Abcam, ab32503, 1 : 1000), rabbit anti-Bcl-2 (Abcam, ab185002, 1 : 500), rabbit anti-AKT (Abcam, ab8805, 1 : 1000), rabbit anti-p-AKT (Abcam, ab8933, 1 : 1000), rabbit anti-glycogen synthase kinase-3 (GSK-3) *β* (Abcam, ab32391, 1 : 1000), and rabbit anti-p-GSK3*β* (Abcam, ab75814, 1 : 1000). After being incubated with a peroxidase-coupled secondary antibody, these bands were scanned with a BioSpectrum Gel Imaging System, respectively (UVP, California, USA).

### 2.7. Quantitative Real-Time PCR Analysis

We used TRIzol to extract total RNA from left ventricles. We used the PrimeScript RT Reagent Kit (#RR036B, TaKaRa, Otsu, Japan) to perform reverse transcriptional reactions. Quantitative real-time PCR was performed using the SYBR® Premix Ex Taq™ II Kit (#RR820DS, TaKaRa). GAPDH was used for normalization [19].

### 2.8. TUNEL and Caspase 3 Activity Assay

To detect cell apoptosis after DOX treatment, terminal deoxynucleotidyl transferase-mediated dUTP nick-end labeling (TUNEL) staining was performed using the In Situ Cell Death Detection Kit (Roche Applied Science) according to the manufacturer's instructions [20]. The activity of caspase 3 was assayed using the Caspase 3 Activity Assay Kit obtained from Beyotime Biotechnology (Beijing, China).

### 2.9. Cell Culture and Treatment

H9c2 cells were obtained from ATCC (CRL-1446) and cultured in DMEM supplemented with 10% FBS and 0.5% penicillin/streptomycin in a humidified atmosphere of 5% CO_2_ and 95% O_2_ at 37°C. To detect DOX-induced cell injury, H9c2 cells were seeded in 96-well plates (density: 1 × 10^5^ cells/ml). After 48 hours, these cells were pretreated with series doses of AA for 4 hours, which were dissolved into 0.1%DMSO. After that, 0.1%DMSO- or AA-pretreated H9c2 cells were subsequently treated with DOX (5 *μ*g/ml) or PBS for 24 hours to detect cell viability using the CCK-8 kit (Dojindo, Rockville, MD). To detect alteration in oxidative markers, H9c2 cells were treated with DOX (5 *μ*g/ml) for 12 hours. To verify the hypothesis that the protection provided by AA was mediated by the activation of AKT, we used adenoviruses carrying sequences encoding a dominant-negative AKT. Briefly, at 48 hours after plating, H9c2 were infected with Ad-dnAKT or Ad-GFP at 50 MOI for 4 hours. After that, H9c2 cells were treated with AA (20 *μ*mol/l) or 0.1% DMSO in the presence of DOX or PBS treatment.

### 2.10. Data Analysis

All data are expressed as the mean ± standard deviation (SD) and were analyzed by SPSS 22.0 software. We used unpaired Student's *t*-test to compare the differences between two groups. In our study, we used one-way analysis of variance (ANOVA) followed by the Tukey post hoc test for multiple group comparisons. A repeated-measures ANOVA was used to examine the alteration in body weight. *P* < 0.05 was considered statistically significant.

## 3. Result

### 3.1. AA Treatment Attenuated DOX-Induced Cardiac Injury

**Figure 1:**
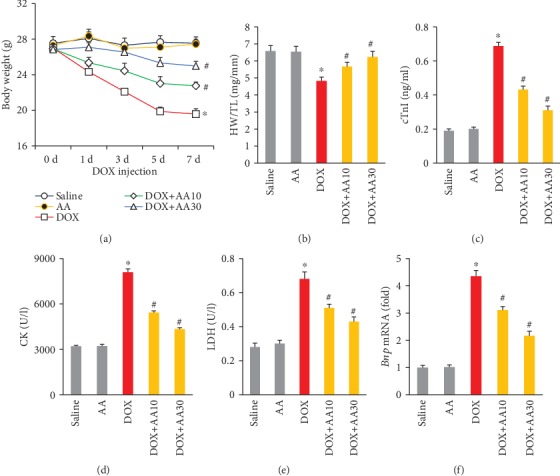
AA attenuated DOX-related cardiac injury in mice. (a) Body weight in the indicated groups (*n* = 12). (b) The ratio of heart weight to tibia length (*n* = 12). (c) The level of cTnI among groups (*n* = 6). (d, e) The plasma CK and LDH in the indicated groups (*n* = 6). (f) The mRNA level of *Bnp* in the hearts (*n* = 6). ^∗^*P* < 0.05 vs. saline group; ^#^*P* < 0.05 vs. DOX group.

Exposure to DOX for 7 days significantly decreased body weight; however, AA-treated mice exhibited more body weight than vehicle-treated mice (Figure 1(a)). DOX treatment resulted in a decreased in the ratio of heart weight to tibia length (HW/TL), and this pathological change was attenuated by AA treatment in a dose-dependent manner (Figure 1(b)). Elevation of plasma cTnI after DOX injection reflects cardiac injury and indicates irreversible cell loss. To evaluate the effect of AA on DOX-induced cardiotoxicity, we detected cTnI levels at three days after DOX injection and found that the release of cTnI induced by DOX injection was largely prevented by oral treatment of AA (Figure 1(c)). The plasma CK and LDH levels in DOX-treated mice were significantly increased compared with those in control groups, and these pathological upregulation could be attenuated by AA in a dose-dependent manner (Figures 1(d) and 1(e)). Further detection of BNP mRNA level suggested that AA also decreased the mRNA level of *Bnp* in DOX-treated mice (Figure 1(f)).

### 3.2. AA Treatment Improved Cardiac Function in Mice with DOX Treatment

**Figure 2:**
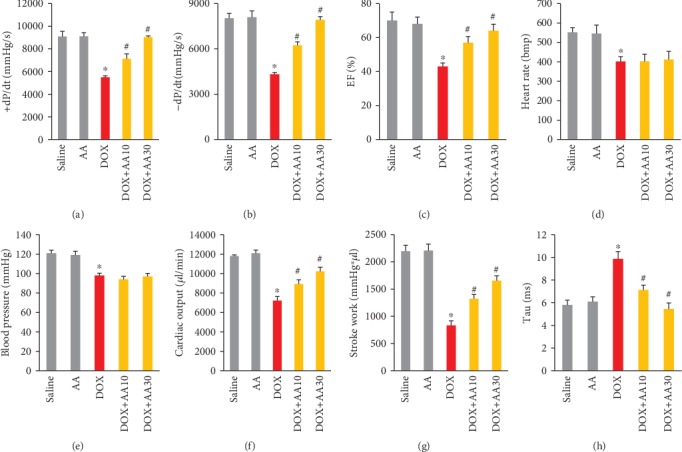
AA treatment improved cardiac function in mice. (a, b) ±dP/dt in the mice (*n* = 8). (c) EF in the indicated groups (*n* = 8). (d, e) Heart rate and blood pressure in the mice (*n* = 8). (f, g) Cardiac output and stroke work (*n* = 8). (h) Tau in the mice (*n* = 8). ^∗^*P* < 0.05 vs. saline group; ^#^*P* < 0.05 vs. DOX group.

Treatment of mice with DOX, 15 mg/kg intraperitoneally, led to a significant decrease in maximum first derivative of ventricular pressure with respect to time (+dP/dt), -dP/dt, and ejection fraction (EF). Oral treatment with AA (10 mg/kg or 30 mg/kg) daily significantly attenuated the DOX-induced changes in ventricular function (Figures 2(a)–2(c)). DOX induced the decrease in heart rate and blood pressure, and there was no difference between DOX and DOX+AA groups in the two parameters (Figures 2(d) and 2(e)). DOX injection induced reductions in cardiac output, stroke work, and prolongation of relaxation time constants (Tau), and these effects were significantly attenuated after AA treatment (Figures 2(f)–2(h)).

### 3.3. AA Treatment Inhibited DOX-Induced Oxidative Injury in the Hearts

**Figure 3:**
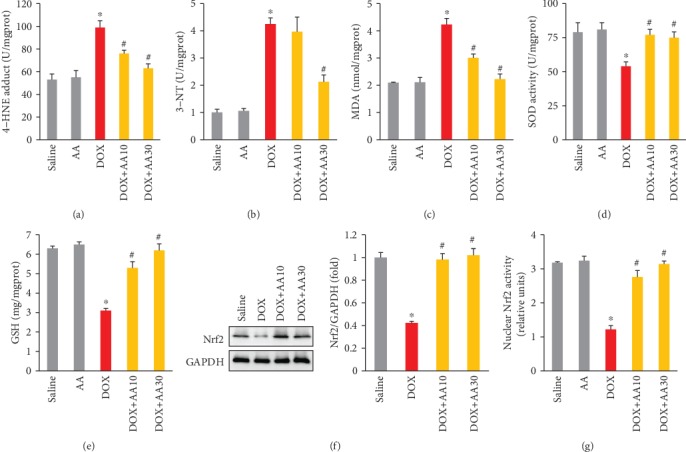
AA inhibited DOX-induced oxidative damage in mice. (a–c) The level of 4-HNE, 3-NT, and MDA in the mice (*n* = 6). (d) The activity of SOD (*n* = 6). (e) The GSH content (*n* = 6). (f, g) The Nrf2 protein expression and activity (*n* = 6). ^∗^*P* < 0.05 vs. saline group; ^#^*P* < 0.05 vs. DOX group.

DOX treatment increased production of 4-HNE in the hearts, which is a highly reactive lipid peroxidation product. To determine the effect of AA on the oxidative injury in the heart after DOX injection, we detected 4-HNE level in the cardiac tissues. ELISA detection revealed that the levels of 4-HNE-adducts were significantly higher in the hearts of DOX-only-treated animals. AA treatment dose-dependently reduced the 4-HNE level in the hearts of DOX-treated animals (Figure 3(a)). AA treatment also decreased the level of 3-NT in the hearts of DOX-treated mice (Figure 3(b)). To further evaluate the lipid peroxidation, we detected the level of MDA and found that AA treatment could significantly decrease the level of MDA in a dose-dependent manner (Figure 3(c)). Of note, we found that AA (30 mg/kg)-treated mice had similar level of MDA as those treated with saline only (Figure 3(c)). DOX injection decreased SOD activity and GSH content; however, these pathological alterations were largely attenuated after AA treatment (Figures 3(d) and 3(e)). As shown in Figure 3(f), the expression levels of cardiac Nrf2 were obviously decreased in DOX groups compared with those in control groups. And AA treatment could restore cardiac Nrf2 to the normal level (Figure 3(f)). DOX treatment significantly lowered cardiac Nrf2 activity, and AA treatment almost increased active Nrf2 in nuclear extracts to the level of those in the hearts of mice with saline only (Figure 3(g)). Next, we examined a number of genes that are known to be regulated by Nrf2. Without DOX injection, AA increased the mRNA levels of *Sod2* and *Gpx4* at basal condition (Table 1). All the examined transcript levels, including *Cat*, *Sod1*, *Sod2*, *Gpx1*, *Gpx4*, *Ho-1*, *Nqo-1*, *Gsta3*, and *Gsta4*, were decreased in the DOX-treated group. The reductions in these genes were largely reversed after AA treatment (Table 1).

### 3.4. AA Treatment Prevented DOX-Induced Cardiac Apoptosis In Vivo

**Figure 4:**
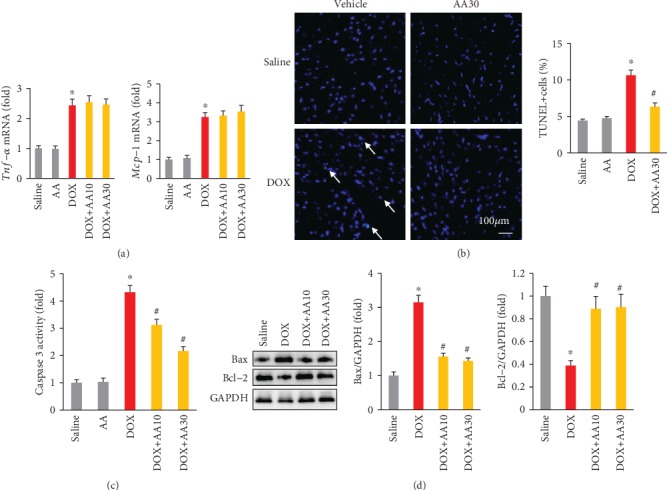
AA reduced cardiac apoptosis in DOX-treated mice. (a) The mRNA levels of inflammatory factors in the hearts (*n* = 6). (b) TUNEL staining (*n* = 6). (c) Caspase 3 activity in the hearts (*n* = 6). (d) The expression of Bax and Bcl-2 in the hearts (*n* = 6). ^∗^*P* < 0.05 vs. saline group; ^#^*P* < 0.05 vs. DOX group.

As inflammation also plays a key role in DOX-mediated cardiac injury, next we detected cardiac inflammatory factors and found that there was no difference in the mRNA levels of *Tnf-α* and *Mcp-1* between DOX and DOX+AA groups (Figure 4(a)). A DOX injection induced an increase in the number of TUNEL-positive cells in mice, and AA treatment could significantly decrease the number of TUNEL-positive cells (Figure 4(b)). Further detection of caspase 3 activity revealed that AA treatment decreased the elevation of caspase 3 activity in DOX-treated mice (Figure 4(c)). Furthermore, AA treatment also significantly attenuated DOX-induced upregulation of Bax, as well as downregulation of Bcl-2, in the hearts (Figure 4(d)).

### 3.5. AA Treatment Attenuated DOX-Induced Oxidative Stress and Cell Death in Cardiomyocytes

**Figure 5:**
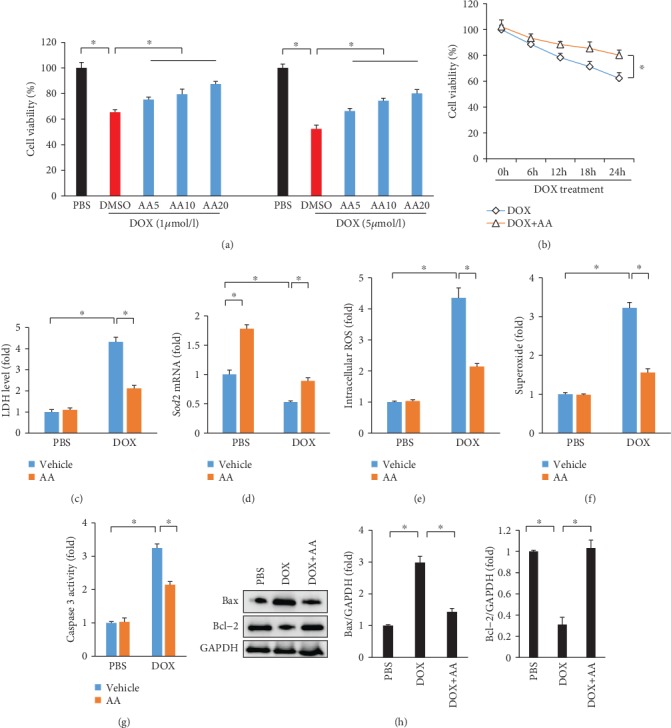
AA improved cell viability and reduced cell injury in vitro. (a, b) Cell viability after DOX treatment (*n* = 6). (c) LDH release after DOX (*n* = 6). (d) The mRNA level of *Sod2* (*n* = 6). (e, f) ROS and superoxide levels (*n* = 6). (g) Caspase 3 activity in the cells (*n* = 6). (h) The protein expression of Bax and Bcl-2. ^∗^*P* < 0.05 compared with the matched control.

To further confirm the role of AA in DOX-mediated cardiomyocytes injury, we used H9c2 cardiomyocytes. Incubation of H9c2 cells with 1 or 5 *μ*mol/l DOX for 24 hours resulted in significant decreases in cell viability, as measured by CCK-8 assay to 65.3 ± 2.1% or 52.3 ± 2.8%, respectively. DOX-induced cell death was prevented by 4 h of pretreatment (followed by constant incubation during the 24 hours of DOX exposure) with AA treatment (DOX 1 *μ*mol/l+AA 20 *μ*mol/l: 87.4 ± 1.9%; DOX 5 *μ*mol/l+AA 20 *μ*mol/l: 80.1 ± 2.0%) (Figure 5(a)). This result was further confirmed by the following finding that AA (20 *μ*mol/l) improved cell viability in DOX-treated cardiomyocytes at different time points (Figure 5(b)). The increased LDH levels in DOX-treated H9c2 cells were suppressed by AA treatment (Figure 5(c)). AA treatment also increased the mRNA level of *Sod2* in cells with DOX administration (Figure 5(d)). Compared with the PBS group, the intracellular ROS level and superoxide in cardiomyocytes in the DOX group was remarkably increased. However, AA treatment markedly decreased the ROS level in DOX-treated H9c2 cells (Figures 5(e) and 5(f)). Next, we detected the alteration in caspase 3 activity and found that AA treatment significantly suppressed the upregulation of caspase 3 activity (Figure 5(g)). AA treatment also suppressed upregulation of Bax and downregulation of Bcl-2 in H9c2 cardiomyocytes with DOX treatment (Figure 5(h)).

### 3.6. AA Exerted Protection against Cardiac Injury via Activation of AKT Signaling Pathway

**Figure 6:**
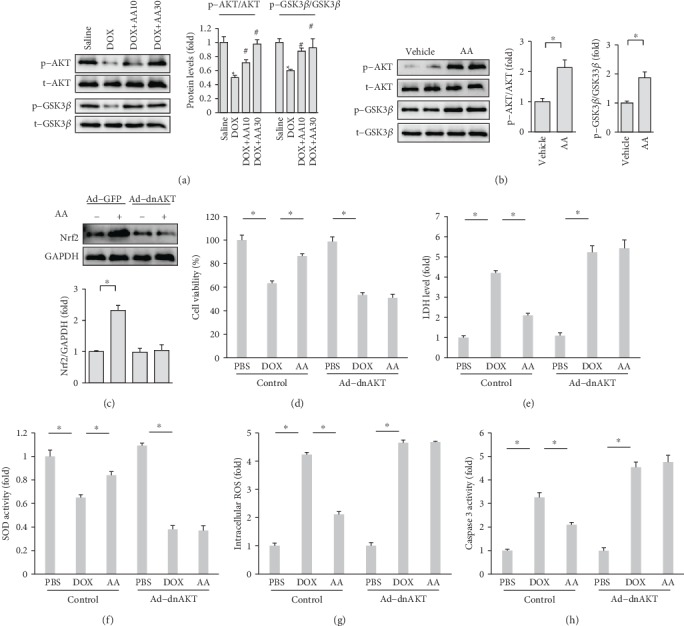
AA provided cardioprotection via the activation of AKT signaling pathway. (a, b) AKT and GSK3*β* in the H9c2 cells (*n* = 6). (c) The protein expression of Nrf2 (*n* = 6). (d) Cell viability in the cells (*n* = 6). (e) LDH release in vitro (*n* = 6). (f) SOD activity after DOX treatment (*n* = 6). (g) ROS production in the cells (*n* = 6). (h) Caspase 3 activity (*n* = 6). ^∗^*P* < 0.05 compared with the matched control.

Previous study demonstrated that AA protected against diabetes-related injury via activation of the AKT signaling pathway [21]. Next, we detected the alteration in the AKT signaling pathway. DOX significantly decreased the phosphorylation of AKT, and AA completely restored this to the normal level (Figure 6(a)). Further detection of downstream target also suggested that AA could promote the activation of the GSK3*β* signaling pathway (Figure 6(a)). To further confirm this, we detected this signaling pathway in vitro and found that AA activated the AKT-GSK3*β* pathway even without any stimuli (Figure 6(b)). To verify the protection of AA against cell injury which was mediated by activation of the AKT signaling pathway, we used adenoviruses to overexpress a plasmid carrying sequences encoding a dominant-negative AKT. AA treatment-induced Nrf2 upregulation was blocked by dominant-negative AKT infection (Figure 6(c)). As shown in Figures 6(d) and 6(e), AA lost its protection in cells infected with Ad-dnAKT, as indicated by cell viability and LDH level. Ad-dnAKT infection also completely abolishes the effect of AA on SOD activity and intracellular ROS by AA administration in vitro (Figures 6(e)–6(g)). AA treatment decreased caspase 3 activity, and this effect was reversed by a dominant-negative AKT (Figure 6(h)).

### 3.7. AA Had No Cardiac Protection in Mice Infected with Ad-dnAKT

**Figure 7:**
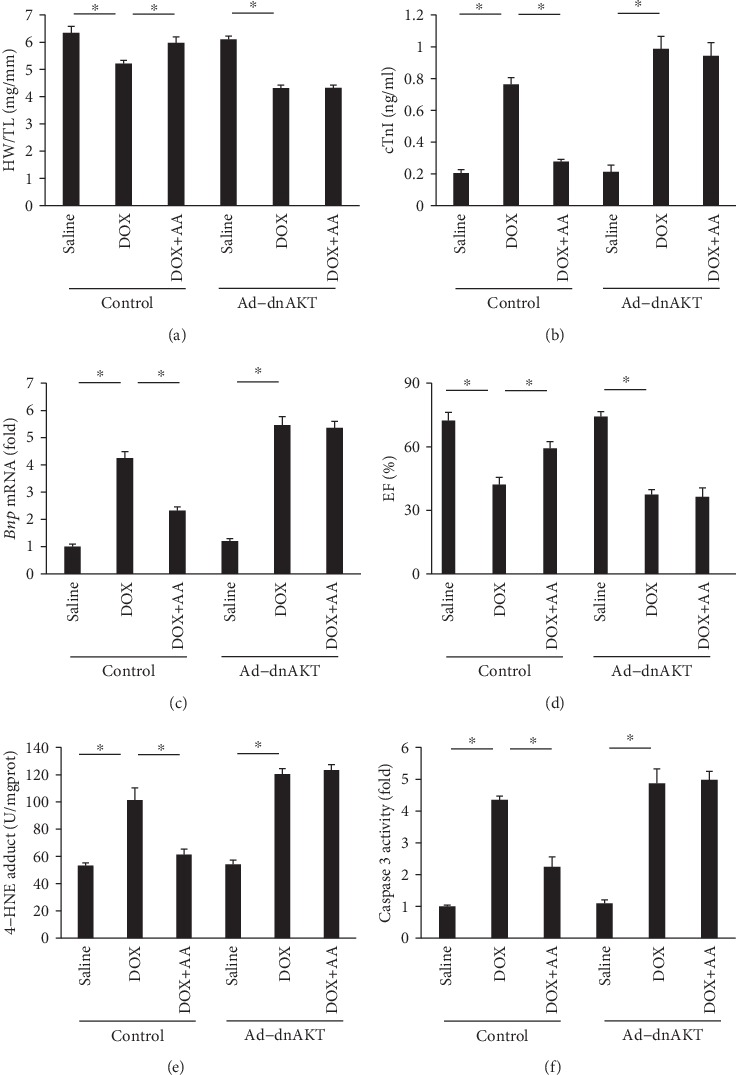
AA lost cardiac protection in the hearts after AKT inactivation. (a) The ratio of heart weight to tibia length (*n* = 10). (b) The level of cTnI among groups (*n* = 6). (c) The mRNA level of *Bnp* in the hearts (*n* = 6). (d) EF (*n* = 8). (e, f) The levels of 4-HNE and caspase 3 activity in the hearts (*n* = 6). ^∗^*P* < 0.05 compared with the matched control.

To further confirm the role of AKT in AA-mediated protection, mice were subjected to intramyocardial injection of Ad-dnAKT. DOX-treated mice infected with Ad-dnAKT showed reversal of the inverse morphological changes during AA treatment, as reflected by HW/TL, cTnI level, *Bnp* mRNA level, EF, 4-HNE content, and caspase 3 activity (Figures 7(a)–7(f)).

## 4. Discussion

In the present study, we investigated the effect of AA on DOX-induced cardiotoxicity. Our results showed that AA treatment protected against DOX-induced cardiac injury, oxidative stress, and cell death without affecting inflammation. AA treatment activated the AKT signaling pathway in vivo and in vitro. AA lost its protection against oxidative stress and cell apoptosis after AKT inhibition. Thus, our study indicated that AA activated the AKT signaling pathway to suppress DOX-related cardiac injury.

Currently, there were no specific strategies to prevent DOX-induced cardiotoxicity. Moreover, heart failure caused by DOX is standardly treated to alleviate symptoms and improve life quality. Finding a drug would be of great clinical significance. Indeed, several chemicals have been evaluated for their ability to attenuate DOX-related cardiac injury, but with little success [22, 23]. Lack of benefit of these drugs was largely attributed to low bioavailability of drugs or secondary reactions with other molecules [24]. Here, we tested AA and found AA obviously prevented DOX-induced cardiac injury, as reflected by increased body weight and HW/TL, and decreased levels of cardiac injury markers, including cTnI, CK, and LDH. Moreover, AA existed in a number of edible vegetables (including brown mustard and spinach) and dietary intake of AA increased their bioavailability in the heart [10, 25]. Additionally, in our study, we did not observe any adverse events in mice with AA (30 mg/kg) daily. Taken together, these data suggested that AA has the potential for the clinical use.

There were a lot of mitochondria which could produce ROS and relatively lower levels of oxidative enzymes, making the hearts more susceptible to oxidative stress. Previous studies have reported that DOX resulted in accumulation of ROS, which stimulated lipid peroxidation and formed highly reactive electrophile 4-HNE [26]. DOX treatment also depleted cardiac GSH levels [27]. In agreement with these studies, we also found that the levels of 4-HNE, 3-NT, and MDA in the hearts were increased. AA inhibited the pathological accumulation of these products. AA also improved SOD activity and increased cardiac GSH content. In vitro, we found that AA decreased the production of ROS and superoxide. Nrf2 is a transcription factor that regulates the expression of antioxidant and detoxification genes [28]. Next, we also found that AA treatment upregulated Nrf2 protein expression and activity in vivo. The reduced oxidative damage caused by AA treatment might partly explain the improved cardiac function in AA-treated mice.

Unexpectedly, we found that AA treatment unaffected the mRNA levels of inflammatory factors, which is inconsistent with a previous study that AA attenuated lipopolysaccharide-induced acute lung injury in mice by inhibiting inflammatory factor production [29]. This discrepancy might be explained by different disease models. Cell loss is one the main reason of the impaired cardiac function in DOX-treated mice. The data in our study indicated that AA treatment reduced cardiac apoptosis in mice and improved cardiomyocyte viability in vitro, which was in line with the previous study [11]. The protection provided by AA against DOX-related cardiac injury was also partly attributed to the prosurvival effect of AA.

Previous study has found that the AKT phosphorylation facilitated the translocation of Nrf2 [30]. It has been found that AA enhanced Nrf2 to protect HepG2 cells from oxidative damage through AKT activation [14]. Here, we found that AA increased phosphorylation of AKT in vivo and in vitro. Using dominant-negative AKT, we found that the activation of Nrf2 by AA was blocked. In line with this finding, we found that the inhibitory effects of AA on oxidative damage and cardiac apoptosis were offset by the infection of Ad-dnAKT. The improvement of cardiac function after AA treatment in DOX-treated animals was also lost after AKT inactivation. These data indicated that the protective effects of AA were mediated by AKT activation. However, there sounds a quite different voice that AA exerted anticancer effect in human ovarian cancer cells via suppression of AKT signaling [31]. This discrepancy could be explained by the different roles of AKT in different models.

In conclusion, we found that our results demonstrated that AA protected against DOX-induced cardiomyopathy via activating AKT signaling, which restored Nrf2 activation and suppressed oxidative damage to improve cardiac function. Our study may offer a new perspective on the treatment of DOX cardiac toxicity.

## Figures and Tables

**Table 1:** The genes that regulated by Nrf2.

Gene name	Control	AA30	DOX	DOX+AA30
*Cat*	0.88 ± 0.12	0.93 ± 0.09	0.32 ± 0.07^∗^	0.73 ± 0.05^#^
*Sod1*	2.16 ± 0.22	2.15 ± 0.34	0.64 ± 0.12^∗^	1.89 ± 0.18^#^
*Sod2*	3.17 ± 0.23	4.76 ± 0.21^∗^	1.14 ± 0.11^∗^	2.67 ± 0.24^#^
*Gpx1*	0.94 ± 0.09	0.89 ± 0.07	0.45 ± 0.04^∗^	0.78 ± 0.08^#^
*Gpx4*	3.58 ± 0.22	4.54 ± 0.13^∗^	2.14 ± 0.34^∗^	3.13 ± 0.46^#^
*HO-1*	0.25 ± 0.04	0.27 ± 0.05	0.09 ± 0.01^∗^	0.18 ± 0.02^#^
*NQO1*	0.58 ± 0.03	0.55 ± 0.05	0.32 ± 0.04^∗^	0.43 ± 0.05^#^
*Gsta3*	0.09 ± 0.01	0.08 ± 0.02	0.04 ± 0.01^∗^	0.07 ± 0.01^#^
*Gsta4*	2.44 ± 0.21	2.54 ± 0.43	1.02 ± 0.11^∗^	2.54 ± 0.12^#^

^∗^ *P* < 0.05 vs. control group; ^#^*P* < 0.05 vs. DOX group.

## Data Availability

The data that support the findings of this study are available from the corresponding author upon reasonable request.
